# The anti-clockwise spiralization of the *linea nigra* sign

**DOI:** 10.31744/einstein_journal/2020AO5432

**Published:** 2020-10-29

**Authors:** Daniel Klotzel, Marina Zamuner, Andrea Maria Novaes Machado, Cristina Tiemi Amadatsu, Adolfo Wenjaw Liao

**Affiliations:** 1 Hospital Israelita Albert Einstein São PauloSP Brazil Hospital Israelita Albert Einstein, São Paulo, SP, Brazil.

**Keywords:** *Linea nigra*, Hyperpigmentation/diagnosis, Skin/pathology, Skin pigmentation, Physiological changes in pregnancy

## Abstract

**Objective:**

To describe the morphology of the supra- and infraumbilical *linea nigra* in puerperal women.

**Methods:**

The study was conducted from September 2017 to April 2018, and included 157 puerperal women admitted for childbirth care at the Obstetrics Department of a public maternity hospital of the city of São Paulo (SP), Brazil. The abdomen of subjects was photographed on the first or second day postpartum, with the patient lying symmetrically in dorsal decubitus at a standardized distance. Contrast was slightly adjusted and the morphological pattern of supra and infraumbilical *linea nigra* in the proximity of the umbilical scar was characterized. The images were independently analyzed by two researchers and only the matching results from both observers were used.

**Results:**

Of the 157 observed cases, 139 (88.5%) images provided concordant results between the two researchers. Excluding 41 cases of absence or poor definition of the *linea nigra*, 98 images were analyzed. Supra- and infraumbilical *linea nigra* were analyzed separately and classified according to three directions (left, center and right of the umbilical scar). The combination of the supra- and infraumbilical images resulted in the formation of nine distinct patterns, being the most prevalent, in primiparous (72.2%) and multiparous women (50.0%), and the authors named as “anticlockwise spiralization of the *linea nigra*”.

**Conclusion:**

The analysis of supra- and infraumbilical *linea nigra* in puerperal women showed a predominance of what the authors named “anti-clockwise spiralization of the *linea nigra* sign”.

## INTRODUCTION

Clinical practice provides an inexhaustible source of observation and valuable knowledge of physiological phenomena, as well as changes leading to pathological situations. After 35 years of professional practice, the senior author of this article noticed what could be a repetitive pattern of the *linea nigra*, the formation of an anti-clockwise spiralization around the umbilical scar. An independent and similar observation was reported by another author more than two decades ago, but the conclusions differ from the present findings.^(^^1^^)^

Hyperpigmentation is the most common physiological skin alteration in pregnancy and is more frequent in women with darker skin.^(^^2^^,^^3^^)^ It is characterized by darkening of areas that are already pigmented (areolae, genitalia, armpits, periumbilical region and inner thighs), melasma and darkening of the linea alba, which leads to the formation of a linear brownish band, along the abdomen midline, called *linea nigra.*^(^^4^^–^^7^^)^

In general, the *linea nigra* is longitudinally located between the pubic symphysis and the umbilical scar, and it may extend up to the xiphoid appendix, although there are some particularities regarding its morphology.^(^^4^^)^ There is very scarce research reporting the details of the pattern of the *linea nigra* in pregnant and puerperal women.

This article reports the observational study of the morphology of the supra- and infraumbilical pattern of the *linea nigra* in its proximity to the umbilical scar, in puerperal women.

## OBJECTIVE

To describe the morphology of the supra- and infraumbilical *linea nigra* in puerperal women.

## METHODS

The study was conducted from September 2017 to April 2018, and included 157 puerperal women admitted for childbirth care at the Obstetrics Department of the *Hospital Municipal de Vila Santa Catarina*, a public maternity hospital of the city of São Paulo (SP), Brazil. After approval by the Research Ethics Committee of the organization (number # 2.233.215, CAAE: 70691317.2.0000.0071), data collection was performed. Subjects were randomly approached during admission for childbirth at the obstetrics ward, regardless of the presence or absence of *linea nigra*. The study was explained, and those who agreed to participate, had their pictures taken after signing an Informed Consent Form. No exclusion criteria were applied at this point. The study followed the principles set forth by the Helsinki Declaration of 1975, as revised in 2013.

Demographic data (age and reported skin color) and number of previous deliveries were collected from hospital records. Puerperal abdomens were photographed, by the same observer on the first or second day postpartum, at the obstetrics ward of the hospital. The patients laid symmetrically in dorsal decubitus, for the photographs to be taken using a Backlit Sony Exmor RS 12 MP (1.22μm), f/2.2 aperture, approximately 30cm away from the patient's abdomen. To ensure the equidistant visualization of the supra- and infraumbilical lines, and the analysis of both in relation to the umbilical scar, the photographs were taken perpendicularly to the abdomen of the patients. Later, contrast was slightly adjusted to allow a better characterization of the morphological pattern of the supra- and infraumbilical *linea nigra* in the proximity of the umbilical scar. The images were independently analyzed by two researchers and only the matching results from both observers were used.

**Figure 1:**
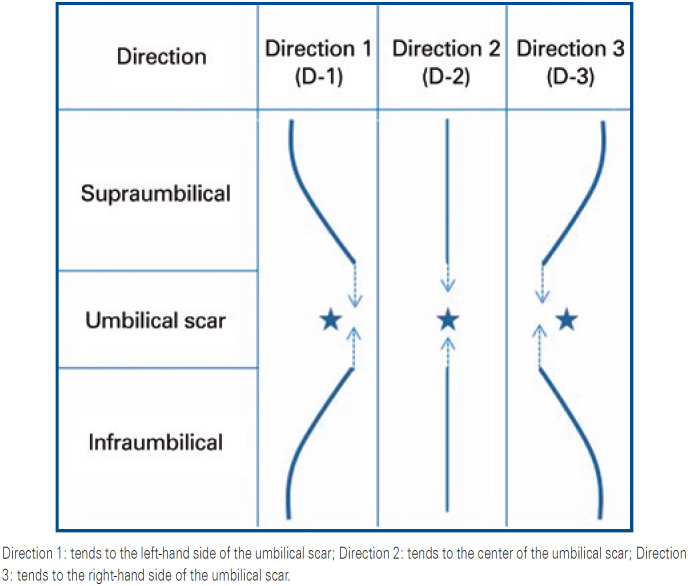
Direction of the *linea nigra* in relation to the umbilical scar

Supra- and infraumbilical *linea nigra* were analyzed separately and classified according to three directions, as shown in figure 1.

**Figure 2:**
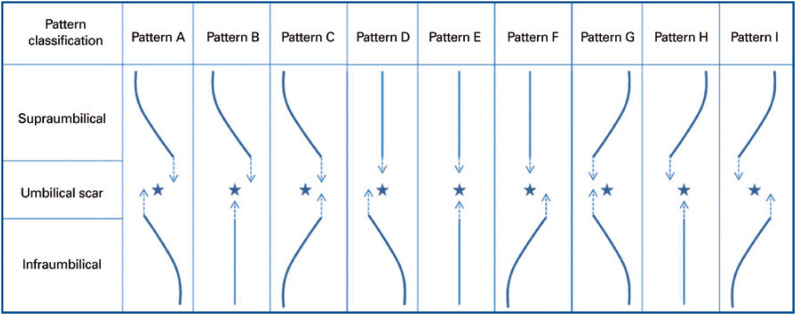
Classification of the supra- and infraumbilical *linea nigra* combination

The supra- and infraumbilical lines were classified in relation to the umbilical scar (to the left, central or to the right of the umbilical scar) and their combinations presented nine figurative patterns (Figure 2).

## RESULTS

Of the 157 observed cases, 139 (88.5%) images provided concordant results between two researchers.

Of the 139 selected images, 41 (29.5%) presented an absent line or a line with poor sharpness; of these, 37 were supraumbilical lines, 17 infraumbilical and 13 both. The highest frequency of supraumbilical images with low sharpness was due to the presence of piercing scarring (27 cases; 72.9%). After the exclusion of these patients, we ended up with 98 quality images to assess the direction of the *linea nigra*.

The age of the study population ranged from 14 to 45 years, mean of 26.9 years. The study included 36 women pregnant for the first time (primiparous, 36.7%), 58 women who had previous pregnancies (multiparous, 59.2%) and four women whose parity information was not available (4.1%) (Table 1).

**Table 1:** Demographic data of the study population

Age		26.9±6.7
Parity, n (%)		
	1	36 (36.7)
	2-3	36 (36.7)
	4-7 prdpregnancies	22 (22.5)
	No information	4 (4.1)
Ethnicity, n (%)		
	Black*	64 (65.3)
	White	31 (31.6)
	No	3 (3.1)

* Brown + black - self-reported. Brazilian Institute of Geography and Statistics.

Among the 94 women with parity information, the predominant directions (which, combined, generated the pattern I) were D-3 for the supra line (73.4%) and D-1 for the infra line (72.3%), and were more prevalent in primiparous (Table 2).

**Table 2:** Direction of the supra- and infraumbilical *linea nigra* according to parity

Direction	Supraumbilical			Infraumbilical		
	Primiparous (%)	Multiparous (%)	Total (%)	Primiparous (%)	Multiparous (%)	Total (%)
D1	3 (8.3)	14 (24.1)	17 (18.1)	29 (80.5)	39 (67.2)	68 (72,3)
D2	0 (0)	8 (13.8)	8 (8.5)	6 (16.7)	6 (10.4)	12 (12,8)
D3	33 (91.7)	36 (62.1)	69 (73.4)	1 (2.8)	13 (22.4)	14 (14,9)
Total	36 (100)	58 (100)	94 (100)	36 (100)	58 (100)	94 (100)

**Figure 3:**
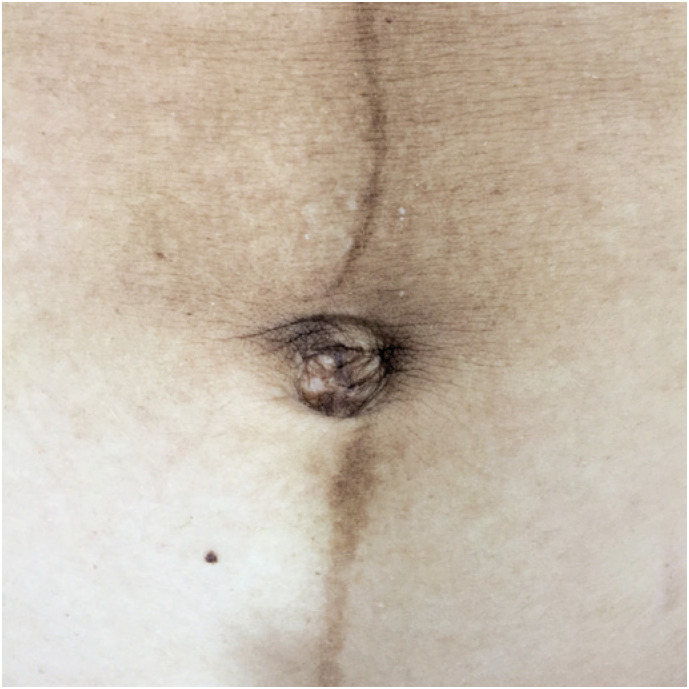
The anti-clockwise spiralization of the *linea nigra* sign

The combination of the supra- and infraumbilical images resulted in the formation of nine distinct patterns (Table 3, Figure 2). Pattern I, which we named “anti-clockwise spiralization of the *linea nigra*” (Figure 3), was the most prevalent pattern in primiparous (26; 72.2%) and in multiparous women (29; 50.0%).

**Table 3:** Distribution of the supra- and infraumbilical *linea nigra* combinations, as per parity and pattern

Pattern	Primiparous (%)	Multiparous (%)	Total (%)
A	0 (0)	6 (10.3)	6 (6.4)
B	0 (0)	1 (1.7)	1 (1.1)
C	3 (8.3)	7 (12.1)	10 (10.6)
D	0 (0)	3 (5.2)	3 (3.2)
E	0 (0)	2 (3.4)	2 (2.1)
F	0 (0)	3 (5.2)	3 (3.2)
G	1 (2.8)	4 (6.9)	5 (5.3)
H	6 (16.7)	3 (5.2)	9 (9.6)
I	26 (72.2)	29 (50.0)	55 (58.5)
Total	36	58	94 (100)

## DISCUSSION

The topic of this article, although intriguing, does not have many academic publications to support it. Beside the mere description of the *linea nigra*, differences in its pattern were first described in 1996, in an article published by researchers from Australia, Beischer et al.^(^^1^^)^ These authors analyzed only the infraumbilical portion of the *linea nigra* and reported a frequency of 13.9% in the studied population. In our study, the frequency of the infraumbilical *linea nigra* was 87.7%. This difference may be related to the study populations, that were diverse in terms of ethnicity and parity, and to the fact that our study was conducted in puerperal women and based on image capture. Another study carried out in four Primary Care Units in the city of Sao Paulo, in 2008, with 123 pregnant women, reported the presence of *linea nigra* in 37.3% of patients, without distinction between the supra- and infraumbilical portions.^(^^8^^)^

Our study, however, was conducted in puerperal women. This choice was based on the hypothesis that, after delivery and consequent decrease of abdominal distension, there would be a higher concentration of skin pigments, allowing a better identification of the presence and morphology of the *linea nigra*.

According to Beischer et al.,^(^^1^^)^ 70% of women presenting *linea nigra* had a shift of this line to the right, along with the displacement of the umbilical scar. This phenomenon was attributed to the tightening of the teres and falciform ligaments due to the increased abdominal volume during gestation. The authors studied only primiparous women at term (>37 weeks) and analyzed the abdomen with women in dorsal decubitus, in cephalad direction, which did not enable an adequate visibility of the supraumbilical portion of the *linea nigra*. In addition, the authors analyzed the *linea nigra* and umbilical scar in relation only to the abdominal midline of the pregnant women. Our analysis was based on photographs taken perpendicularly to the abdomen of the patients, which allowed the equidistant visualization of the supra- and infraumbilical lines, and the analysis of both in relation to the umbilical scar. With this method, we avoided biases, such as parallax effects, which may have impaired the analysis of Beischer et al.^(^^1^^)^

Importantly, all images were analyzed by two separate researchers, and we only used those in which the rotation direction was consistent between the two researchers. By doing this we minimized the bias associated with observer-dependent subjectivity.

Beischer et al.,^(^^1^^)^ analyzed only primiparous women, since they believed previous pregnancies could cause flaccidity of the abdomen, being a confounding factor on the results. In fact, we observed a greater dispersion of the patterns in multiparous women (50.0% of pattern I) than in primiparous (72.2% of pattern I), possibly due to a “blurring” effect on the *linea nigra* after several pregnancies. However, other causes, such as obesity and the presence of stretch marks (also a result of multiparity), should be considered.

The authors suggest that the higher occurrence of pattern I, which we named “anti-clockwise spiralization of the *linea nigra* sign”, may be due to the 270° anti-clockwise rotation of the mesentery,^(^^9^^)^ which occurs during the return of the physiological omphalocele to the abdominal cavity, around the 10^th^ week of embryonic life.^(^^10^^)^

## CONCLUSION

The analysis of the supra and infraumbilical *linea nigra* in puerperal women showed a predominance of what the authors named “the anti-clockwise spiralization of the *linea nigra* sign”.
