# Supplementary material for: The anti-clockwise spiralization of the linea nigra sign
Source: Einstein (Sao Paulo). 2020 Oct 29;18:eAO5432. doi: 10.31744/einstein_journal/2020AO5432 (PMC7607916; doi:10.31744/einstein_journal/2020AO5432)
Supplement: Translation

# Translation: O sinal do espiralamento anti-horário da *linea nigra*

**Authors:** Daniel Klotzel, Marina Zamuner, Andrea Maria Novaes Machado, Cristina Tiemi Amadatsu, Adolfo Wenjaw Liao

**Affiliations:** 1 São PauloSP Brasil Hospital Israelita Albert Einstein, São Paulo, SP, Brasil.

**Keywords:** *Linea nigra;*, Hiperpigmentação/diagnóstico, Pele/patologia, Pigmentação da pele, Alterações fisiológicas na gravidez

## Abstract

**Objetivo:**

Descrever a morfologia da *linea nigra* supra e infraumbilical em puérperas.

**Métodos:**

O estudo foi realizado no período de setembro de 2017 a abril de 2018 e incluiu 157 puérperas admitidas para o parto no Serviço de Obstetrícia de uma maternidade pública da cidade de São Paulo (SP). O abdome das pacientes foi fotografado no primeiro ou segundo dia pós-parto, com a paciente deitada simetricamente em decúbito dorsal a uma distância padronizada. O contraste foi ligeiramente ajustado, e o padrão morfológico da *linea nigra* supra e infraumbilical na proximidade da cicatriz umbilical foi caracterizado. As imagens foram analisadas independentemente por dois pesquisadores, e apenas os resultados concordantes dos dois observadores foram utilizados.

**Resultados:**

Dos 157 casos observados, 139 (88,5%) imagens apresentaram resultados concordantes entre os dois pesquisadores. Excluindo 41 casos de ausência ou má definição da *linea nigra*, 98 imagens foram analisadas. As *linea nigra* supra e infraumbilicais foram analisadas separadamente e classificadas de acordo com três direções (esquerda, centro e direita da cicatriz umbilical). A combinação das imagens supra e infraumbilicais resultou na formação de nove padrões distintos, sendo os mais prevalentes nas primíparas (72,2%) e multíparas (50,0%), o que os autores denominaram “espiralamento anti-horário da *linea nigra*”.

**Conclusão:**

A análise das *linea nigra* supra e infraumbilical em puérperas mostrou predominância do que os autores denominaram “sinal do espiralamento anti-horário da *linea nigra*”.

## INTRODUÇÃO

A prática clínica é uma fonte importante de geração de conhecimento, por propiciar fonte inesgotável de observação dos fenômenos fisiológicos, bem como alterações em situações patológicas. Após 35 anos de prática profissional, o autor principal deste artigo constatou algo que poderia ser um padrão repetitivo da *linea nigra*, a formação de um espiralamento no sentido anti-horário em torno da cicatriz umbilical. Uma observação independente e semelhante foi reportada por outro autor, há mais de duas décadas, mas as conclusões foram diferentes dos achados deste estudo.^(^^1^^)^

A hiperpigmentação é a alteração cutânea fisiológica mais comum na gestação e mais frequente em mulheres de pele mais escura.^(^^2^^,^^3^^)^ Caracteriza-se pelo escurecimento de áreas já pigmentadas (aréolas, genitais, axilas, região periumbilical e face interna das coxas), melasma e escurecimento da linha alba, que leva à formação de uma faixa linear acastanhada, ao longo da linha média do abdome, denominada *linea nigra*.^(^^4^^–^^7^^)^

Em geral, a *linea nigra* localiza-se longitudinalmente entre a sínfise púbica e a cicatriz umbilical, e pode se estender até o processo xifoide, mas há algumas particularidades sobre sua morfologia.^(^^4^^)^ Há poucos estudos descrevendo em detalhes o padrão da *linea nigra* em gestantes e puérperas.

Este artigo reporta um estudo observacional da morfologia do padrão supra e infraumbilical da *linea nigra* na proximidade da cicatriz umbilical em puérperas.

## OBJETIVO

Descrever a morfologia da *linea nigra* supra e infraumbilical em puérperas.

## MÉTODOS

O estudo foi realizado no período de setembro de 2017 a abril de 2018 e incluiu 157 puérperas admitidas para o parto no Serviço de Obstetrícia do Hospital Municipal de Vila Santa Catarina, uma maternidade pública da cidade de São Paulo (SP). Após a aprovação pelo Comitê de Ética em Pesquisa da instituição (número 2.233.215, CAAE: 70691317.2.0000.0071), foi realizada a coleta de dados. As participantes foram abordadas aleatoriamente durante a internação para o parto na enfermaria de obstetrícia, independentemente da presença ou ausência da *linea nigra*. O estudo foi explicado, e aquelas que concordaram em participar assinaram um Termo de Consentimento Livre e Esclarecido permitindo a captura de imagens. Nenhum critério de exclusão foi aplicado neste momento. O estudo seguiu os princípios estabelecidos na Declaração de Helsinki de 1975, conforme a revisão de 2013.

Dados demográficos (idade e cor da pele reportada) e o número de partos anteriores foram coletados dos registros hospitalares. O abdome das puérperas foi fotografado pelo mesmo observador no primeiro ou segundo dia pós-parto, na enfermaria de obstetrícia do hospital. As fotos foram obtidas com as pacientes deitadas simetricamente, em decúbito dorsal, com uma câmera Sony Exmor RS 12 MP (1,22μm) com retroiluminação, abertura f/2.2, a cerca de 30cm do abdome da paciente. As fotografias foram tiradas perpendicularmente ao abdome das pacientes, permitindo a visualização equidistante das linhas supra e infraumbilicais e a análise de ambas em relação à cicatriz umbilical. Depois, o contraste foi ligeiramente ajustado para permitir uma melhor caracterização do padrão morfológico da *linea nigra* supra e infraumbilical na proximidade da cicatriz umbilical. As imagens foram analisadas independentemente por dois pesquisadores, e foram utilizados apenas os resultados congruentes nas avaliações individuais.

As *linea nigra* supra e infraumbilical foram analisadas separadamente e classificadas de acordo com três direções, conforme mostrado na figura 1.

**Figura 1:** Direção da *linea nigra* em relação à cicatriz umbilical

As linhas supra e infraumbilical foram classificadas em relação à cicatriz umbilical (à esquerda, ao centro ou à direita da cicatriz umbilical), e suas combinações apresentaram nove padrões figurativos (Figura 2).

**Figura 2:** Classificação da combinação das *linea nigra* supra- e infraumbilical

## RESULTADOS

Dos 157 casos observados, 139 (88,5%) imagens apresentaram resultados concordantes entre os dois pesquisadores.

Das 139 imagens selecionadas, 41 (29,5%) apresentaram ausência ou má definição da linha; destas, 37 eram linhas supraumbilicais, 17 infraumbilicais e 13 ambas. A frequência mais alta de imagens supraumbilicais com má definição da linha decorreu da presença de cicatrizes de *piercing* (27 casos; 72,9%). Após a exclusão dessas pacientes, restaram 98 imagens de boa qualidade para avaliação da direção da *linea nigra*.

A idade da população do estudo variou de 14 a 45 anos, com média de 26,9 anos. O estudo incluiu 36 mulheres na primeira gestação (primíparas; 36,7%), 58 com gestações anteriores (multíparas; 59,2%) e quatro sem informações de paridade disponíveis (4,1%) (Tabela 1).

**Tabela 1:** Dados demográficos da população do estudo

Idade		26,9±6,7
Paridade, n (%)		
	1	36 (36,7)
	2-3	36 (36,7)
	4-7	22 (22,5)
	Sem informação	4 (4,1)
Etnia, n (%)		
	Negra*	64 (65,3)
	Branca	31 (31,6)
	Sem	3 (3,1)

* Parda + negra autorreportada. Instituto Brasileiro de Geografia e Estatística.

Dentre as 94 puérperas com informações de paridade, as direções predominantes (que, combinadas, geraram o padrão I) foram D-3 para a linha supra (73,4%) e D-1 para a linha infra (72,3%), que foram mais prevalentes em primíparas (Tabela 2).

**Tabela 2:** Direção da *linea nigra* supra- e infraumbilical de acordo com a paridade

Direção	Supraumbilical			Infraumbilical		
	Primíparas (%)	Multíparas (%)	Total (%)	Primíparas (%)	Multíparas (%)	Total (%)
D1	3 (8,3)	14 (24,1)	17 (18,1)	29 (80,5)	39 (67,2)	68 (72,3)
D2	0 (0)	8 (13,8)	8 (8.5)	6 (16,7)	6 (10,4)	12 (12,8)
D3	33 (91,7)	36 (62,1)	69 (73,4)	1 (2,8)	13 (22,4)	14 (14,9)
Total	36 (100)	58 (100)	94 (100)	36 (100)	58 (100)	94 (100)

A combinação das imagens supra e infraumbilicais resultaram na formação de nove padrões distintos (Tabela 3, Figura 2). O padrão I, denominado “espiralamento anti-horário da *linea nigra*” (Figura 3), foi o mais prevalente em primíparas (26; 72,2%) e em multíparas (29; 50,0%).

**Tabela 3:** Distribuição das combinações das *linea nigra* supra e infraumbilical, de acordo com a paridade e o padrão

Padrão	Primíparas (%)	Multíparas (%)	Total (%)
A	0 (0)	6 (10,3)	6 (6,4)
B	0 (0)	1 (1,7)	1 (1,1)
C	3 (8,3)	7 (12,1)	10 (10,6)
D	0 (0)	3 (5,2)	3 (3,2)
E	0 (0)	2 (3,4)	2 (2,1)
F	0 (0)	3 (5,2)	3 (3,2)
G	1 (2,8)	4 (6,9)	5 (5,3)
H	6 (16,7)	3 (5,2)	9 (9,6)
I	26 (72,2)	29 (50,0)	55 (58,5)
Total	36	58	94 (100)

**Figura 3:** O sinal do espiralamento anti-horário da *linea nigra*

## DISCUSSÃO

Embora o objeto deste artigo seja intrigante, não há muitas publicações acadêmicas que o corroborem. Além da simples descrição da *linea nigra*, as diferenças de padrões foram descritas pela primeira vez em 1996, em um artigo publicado por Beischer et al.,^(^^1^^)^ pesquisadores da Austrália. Os autores analisaram apenas a porção infraumbilical da *linea nigra* e relataram frequência de 13,9% na população estudada. Em nosso estudo, a frequência da *linea nigra* infraumbilical foi de 87,7%. Essa diferença pode ter relação com a população dos dois estudos, que é diversa em termos de etnia e paridade, e o fato de nosso estudo ter sido realizado em puérperas e com base na captura de imagens. Outro estudo realizado em quatro Unidades Básicas de Saúde, na cidade de São Paulo, em 2008, com 123 gestantes, relatou a presença da *linea nigra* em 37,3% das pacientes, sem distinção entre as porções supra e infraumbilical.^(^^8^^)^

Nosso estudo, no entanto, foi conduzido em puérperas. Essa escolha baseou-se na hipótese de que, após o parto e a consequente diminuição da distensão abdominal, haveria maior concentração de pigmentos cutâneos, facilitando a identificação da presença e da morfologia da *linea nigra*.

De acordo com Beischer et al.,^(^^1^^)^ 70% das mulheres com *linea nigra* apresentavam desvio dessa linha para a direita, além de deslocamento da cicatriz umbilical. Esse fenômeno foi atribuído ao estiramento dos ligamentos redondo e falciforme, devido ao aumento do volume abdominal durante a gestação. Os autores estudaram somente primíparas próximo ao termo (>37 semanas) e analisaram os abdomes com as mulheres deitadas em decúbito dorsal, do ponto de vista podocefálico, o que não proporcionou visibilidade adequada da porção supraumbilical da *linea nigra*. Além disso, os autores analisaram a *linea nigra* e a cicatriz umbilical em relação apenas à linha média abdominal da gestante. Nossa análise baseou-se em fotografias realizadas perpendicularmente ao abdome das pacientes, o que permitiu a visualização equidistante das linhas supra e infraumbilicais, e a análise de ambas em relação à cicatriz umbilical. Com esse método, evitamos vieses como, por exemplo, efeitos de paralaxe, que podem ter prejudicado a análise de Beischer et al.^(^^1^^)^

Muito importante também, todas as imagens foram analisadas separadamente por dois pesquisadores, e usamos somente aquelas cuja direção de rotação foi concordante entre os dois observadores. Dessa forma, minimizou-se o viés associado à subjetividade dependente do observador.

Beischer et al.,^(^^1^^)^ analisaram apenas primíparas, pois acreditavam que gestações anteriores poderiam causar flacidez abdominal, um possível fator de confusão dos resultados. De fato, observamos uma maior dispersão dos padrões em multíparas (50,0% do padrão I) do que em primíparas (72,2% do padrão I), possivelmente devido a um efeito de “borramento” da *linea nigra* após múltiplas gestações. No entanto, outras causas, como obesidade e presença de estrias (que também resultam da multiparidade) devem ser consideradas.

Os autores sugerem que a maior ocorrência do padrão I, denominado “espiralamento da *linea nigra*”, pode ser explicado pela rotação anti-horária de 270° do mesentério,^(^^9^^)^ que ocorre durante o retorno da onfalocele fisiológica à cavidade abdominal, próximo à 10ª semana de vida embrionária.^(^^10^^)^

## CONCLUSÃO

A análise das *linea nigra* supra e infraumbilical em puérperas mostrou predominância do que os autores denominaram “sinal do espiralamento anti-horário da *linea nigra*”.
